# Modeling the Ebola zoonotic dynamics: Interplay between enviroclimatic factors and bat ecology

**DOI:** 10.1371/journal.pone.0179559

**Published:** 2017-06-12

**Authors:** Javier Buceta, Kaylynn Johnson

**Affiliations:** 1 Department of Chemical and Biomolecular Engineering, Lehigh University, Bethlehem, PA, 18015, United States of America; 2 Bioengineering Program, Lehigh University, Bethlehem, PA, 18015, United States of America; Shanxi University, CHINA

## Abstract

Understanding Ebola necessarily requires the characterization of the ecology of its main enzootic reservoir, i.e. bats, and its interplay with seasonal and enviroclimatic factors. Here we present a SIR compartmental model where we implement a bidirectional coupling between the available resources and the dynamics of the bat population in order to understand their migration patterns. Our compartmental modeling approach and simulations include transport terms to account for bats mobility and spatiotemporal climate variability. We hypothesize that environmental pressure is the main driving force for bats’ migration and our results reveal the appearance of sustained migratory waves of Ebola virus infected bats coupled to resources availability. Ultimately, our study can be relevant to predict hot spots of Ebola outbreaks in space and time and suggest conservation policies to mitigate the risk of spillovers.

## Introduction

Zoonoses constitute 75% of emerging infectious diseases and pose a significant threat to public health [1, 2]. In particular, the 2014 Ebola epidemic in West Africa has been the largest registered ever: as of December 2016 around 28,000 probable human cases with ∼75% mortality rates in laboratory confirmed patients [3]. In addition, Ebola virus (EV) decimates the great ape population, thus posing a conservation hazard, it represents a major threat worldwide through the importation of infections and its possible misuse as biological weapon [4], and, altogether, has dramatic economic [5], and humanitarian [6] consequences. Therefore, despite the promising advances to find a vaccine [7, 8], understanding the factors and mechanisms underlying Ebola outbreaks and spillovers and developing predictive tools to prevent them are of major interest.

The 2014 EV strain in West Africa has been identified as Zaire’s [9]. Notably, this strain originates thousand of miles away, in Central Africa. The source of the outbreak cannot be ascribed to the mobility of infected humans from Central Africa [10] but to a single zoonotic transmission event in Guinea: a human-bat contact [11].

Evidence supports the idea that bats are the main EV reservoir regardless of the large diversity of EV hosts [12]. First, EV has been detected in tree-roosting bats [13, 14]. Second, inoculation of the virus has revealed that bats can survive EV infection [15–17]. Third, novel filovirus have been only found in bats [18]. Fourth, there is a demonstrated connection of Ebola outbreaks with direct exposure to bats [19]. Finally, as for the expansion of the EV zoonotic niche, satellite telemetry has shown that bats are able to migrate thousands of kilometers annually [20]. Consequently, understanding the Ebola problem requires, on one hand, the characterization of the ecology of its main enzootic reservoir, i.e. bats, and, on the other hand, its interplay with seasonal and enviroclimatic factors [21–26] that drive and shape the bat migration patterns.

During the past years the Ebola modeling efforts have been intense [27–33]. However, these have traditionally focused on human epidemiology (the effect). Here, by focusing on the ecology and its interplay with the environmental conditions (the cause), we aim at shifting the current paradigm. In particular, we explore the feedback between environmental pressure and the dynamics of the EV enzootic niche. Our results show that a bidirectional interplay—i.e., how resources condition the population dynamics and, simultaneously, how the existing population conditions the available resources—may contribute to the expansion of the Ebola zoonotic niche.

The paper is organized as follows. In the Methods section we introduce and characterize theoretically an Ebola zoonotic compartmental model that accounts for the dynamics of the bat population. In the Results section we show by means of numerical simulations how resources variability and seasonality drives bat migration and EV spreading. Finally, the implications and the main conclusions of our research are detailed in the Discussion section.

## Methods

### Ebola zoonotic compartmental model

**Fig 1:**
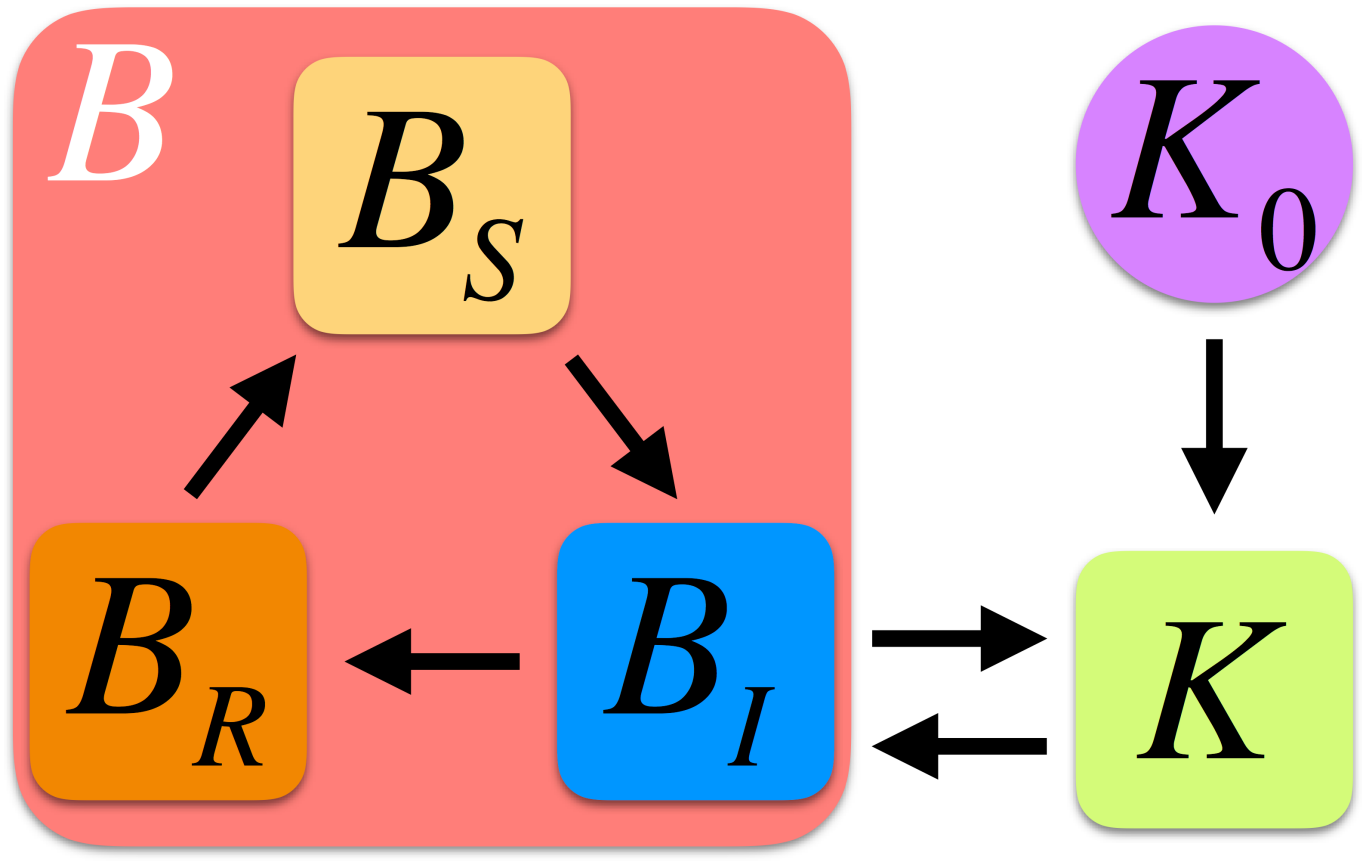
Schematic representation of the compartmental model for the zoonotic dynamics of Ebola virus. Our model considers three possible states for the bats, *B*, population: susceptible (*B*_*S*_), infected (*B*_*I*_), and recovered (*B*_*R*_). The bats population conditions the available resources represented effectively by means of the carrying capacity *K* and, likewise, those resources conditions the size of the bats population. The available resources depends on a “bare” carrying capacity, *K*_0_, that is function of climatic and other environmental factors.

Here we propose a SIR (Susceptible-Infected-Recovered) compartmental zoonotic model for bats. Fig 1 summarizes our modeling approach. Notice that on top of susceptible, *B*_*S*_, infected, *B*_*I*_, states we also consider a recovered (from infection) state, *B*_*R*_. Our hypothesis is based on data about the dynamics of filovirus infection on bats: most of the infected individuals are older juveniles [23] and bats can survive infection [15–17]. A reasonable conjecture to explain the demography of infection is by invoking recovery. During the recovery phase we assume that bats cannot get infected and/or transmit the disease (see Discussion).

In our model we consider the following processes to account for the dynamics of the bat population and the EV infection: birth (rate *b*_*K*_), death (rate *c*), competition for resources (*K*), EV transmission (rate *e*), recovery from infection (rate *d*), and retrieval to the susceptible state from a recovery state (rate *f*). We notice that the obtained analytical results are valid in case *d* = *f* = 0, that is, when the recovery state is suppressed. As detailed below, we also explore the mobility of bats by means of diffusive terms, *D*_*K*_. In addition, we consider an equation for the dynamics of resources, *K* (see Fig 1). By overlooking the mobility terms for the time being, our model for the time evolution of the density of bats in different states reads,

$$\frac{\partial B_{S}}{\partial t}=b_{K}\left(B_{S}+\lambda B_{I}+B_{R}\right)-cB_{S}-\left|b_{K}-c\right|\frac{B_{S}B}{K}-eB_{S}B_{I}+fB_{R}$$ (1)

$$\frac{\partial B_{I}}{\partial t}=\left(1-\lambda \right)b_{K}B_{I}-cB_{I}-\left|b_{K}-c\right|\frac{B_{I}B}{K}+eB_{S}B_{I}-dB_{I}$$ (2)

$$\frac{\partial B_{R}}{\partial t}=-cB_{R}-\left|b_{K}-c\right|\frac{B_{R}B}{K}+dB_{I}-fB_{R}$$ (3)

$$\frac{\partial K}{\partial t}=-\gamma B+r\left(K_{0}-K\right)$$ (4)

where *B* = *B*_*S*_ + *B*_*I*_ + *B*_*R*_ stands for the total population of bats. Bats mobility is considered in our modeling approach by including in the r.h.s. of Eqs (1)–(3) terms of the form +*D*_*K*_∇^2^ *B*_*Z*_, where $\nabla^{2}=\frac{\partial^{2}}{\partial x^{2}}+\frac{\partial^{2}}{\partial y^{2}}$ and *Z* stands for *S*, *I*, or *R*. That is, we consider the simplest form of non-directed mobility, i.e. diffusion. Other forms of transport are certainly possible, e.g. convective terms, but those require additional hypotheses about the causes driving bats migration.

According to the experimental data, EV infection does not modify bats’ physiology. Thus, we assume that the birth and death rates (or the mobility coefficient) do not depend on the state of infection. Yet, as detailed below, we assume that the birth rate and the mobility coefficient depend on the available resources, *K*. Such functional dependence (see details below) effectively summarizes the effect of the environmental pressure in our model. The parameter λ ∈ [0, 1] reflects the lack of information about the possibility that bats are born either EV free, λ = 1, or infected, λ = 0. The demographic data about Marburg filovirus infection [23] indicates that most of the infected individuals are older juveniles and not newborns. Consequently, the former possibility, λ = 1, seems to be the more plausible. Note that the absolute value, |*b*_*K*_ − *c*|, in the equations is required to make the Verhulstian quadratic competition term, ∼*B*^2^, always negative regardless the sign of the growth rate, *a*_*K*_ = *b*_*K*_ − *c*. As for the equation for the resources (carrying capacity), *γ* stands for the rate of depletion of resources by the bats, and *r* accounts for the rate at which the resources naturally return to their “bare” value, *K*_0_. The latter corresponds to the value of the carrying capacity in the absence of bats.

### Simulation scheme and parameter values

Our numerical simulations use the FTCS scheme [34] in an hexagonal lattice. Each hexagonal tile represents a surface of 25 *km*^2^ (unit length: *l*_*c*_ = 5 *km*) and the temporal time step is *t*_*c*_ = 1 *day*. Our simulations span a surface of 2.5 ⋅ 10^4^ *km*^2^ and 10 years such that a) the simulated spatial domain is large enough to justify the spatial variation of resources, and b) several (annual) seasonality periods are included to avoid transient effects due to initial conditions.

The value of the parameters are summarized in Table 1. We try to implement values as realistic as possible. Yet, we must point out that most parameters either have not been measured and/or their characterization has been made for different bat species under different conditions. In any case, the ultimate objective of this study is not to fit factual data about EV infection but to propose a mechanism to explain the interplay between environmental conditions, EV infection and the bat migratory dynamics.

**Table 1:** Parameter values used in numerical simulations.

	Parameter	Units	Value
Birth rate [35]	*b*_1_	$t_{c}^{-1}$	365^−1^
Birth rate	*b*_2_	$t_{c}^{-1}$	0
Death rate [35]	*c*	$t_{c}^{-1}$	(15 ⋅ 365)^−1^
Recovery rate [23]	*d*	$t_{c}^{-1}$	(2 ⋅ 365)^−1^
Infection rate [16, 23, 36, 37]	*e*	$l_{c}^{2}/\left(bat\cdot t_{c}\right)$	2.5 ⋅ 10^−6^
Retrieval rate	*f*	$t_{c}^{-1}$	(2 ⋅ 365)^−1^
Env. relaxational rate	*r*	$t_{c}^{-1}$	365^−1^
Bats consumption rate	*γ*	$t_{c}^{-1}$	365^−1^
Diffusion rate [20, 38]	*D*	$l_{c}^{2}/t_{c}$	25
Bare carrying capacity [39, 40]	〈*K*_0_〉	$bats/l_{c}^{2}$	3 ⋅ 10^3^
Env. pressure carrying capacity	K*	$bats/l_{c}^{2}$	2 ⋅ 10^3^
Seasonal variation coeff.	Γ	−	25%
Seasonal temporal frequency	*ω*	$t_{c}^{-1}$	2*π*/365

## Results

### The interplay between resources and the zoonotic population buffers EV infection

In order to analyze the role played by the interplay between resources and the bat population we analyze first the difussionless case, *D*_*K*_ = 0. By adding Eqs (1)–(3) the equations for the total bat population, *B*, and the resources reduce to,

$$\frac{\partial B}{\partial t}=a_{K}B-\left|a_{K}\right|\frac{B^{2}}{K}$$ (5)

$$\frac{\partial K}{\partial t}=-\gamma B+r\left(K_{0}-K\right)$$ (6)

That is, a logistic growth equation combined with a relaxational dynamics for the resources.

We estimate the onset of infection using two different, complementary, approaches. On one hand, we compute the basic reproduction number, $R_{0}$ using the next generation method [41–45]. Eqs (1)–(4) has a single, non-trivial (*a*_*K*_ > 0), infection-free stationary solution: $\left\{K^{st},B_{S}^{st},B_{I}^{st},B_{R}^{st}\right\}=\left\{\mu K_{0},\mu K_{0},0,0\right\}$, where $\mu =\frac{1}{1+\gamma /r}$. Thus, the equation that describes the dynamics of infection if all bats are infection-free reads [41],

$$\frac{\partial B_{I}}{\partial t}=\left(F-V\right)B_{I}$$ (7)

$$V=d+\lambda b_{K}-a_{K}$$ (8)

$$F=e\mu K_{0}-a_{K}$$ (9)

The first term on the l.h.s. of Eq (7) collects the *transmissions* terms and the second one the *transitions* terms such that [41],

$$\begin{array}{c} R_{0}=\frac{F}{V}=\frac{e\mu K_{0}-a_{K}}{d+\lambda b_{K}-a_{K}} \end{array}$$ (10)

The above expression is valid if *a*_*K*_ = *b*_1_ − *c* > 0 otherwise, if *a*_*K*_ = *b*_2_ − *c* < 0 then the stationary state reads $\left\{K^{st},B_{S}^{st},B_{I}^{st},B_{R}^{st}\right\}=\left\{K_{0},0,0,0\right\}$ and consequently $R_{0}=0$. The infection spreads in the population if $R_{0}>1$ and dies out if $R_{0}<1$. The condition that sets the boundary that separates these behavior is given by $R_{0}=1$ that reduces to $e=e_{c}=\frac{d+\lambda b_{1}}{\mu K_{0}}$. If *e* > *e*_*c*_ then $R_{0}>1$.

Alternatively, the threshold condition for the propagation of the infection can be obtained by means of the Jacobian method [41], i.e. a linear stability analysis [46]. Eqs (5) and (6) has two possible stationary states (attractors): {*K*^*st*^, *B*^*st*^}: {*μK*_0_, *μK*_0_} and {*K*_0_, 0}. By estimating the characteristic polynomial of the Jacobian and implementing the Routh–Hurwitz stability conditions (negative eigenvalues), we conclude that the former attractor is stable is *a*_*K*_ > 0 whereas {*K*_0_, 0} is stable if *a*_*K*_ < 0. Thus, if the growth rate is positive, *b*_*K*_ = *b*_1_ > c, the carrying capacity relaxes to a value *μK*_0_ < *K*_0_ that determines the maximum density of bats that can be maintained by the resources when taking into account consumption. On the other hand, if the growth rate is negative, *b*_*K*_ = *b*_2_ < c, the bat population is extinguished and the resources relax to their bare value, *K*_0_.

The aforementioned attractors can be split into a higher dimensional phase space in terms of the different states. Thus, Eqs (1)–(4) have three attractors, $\left\{K^{st},B_{S}^{st},B_{I}^{st},B_{R}^{st}\right\}$, with physical meaning: {*K*_0_, 0, 0, 0}, that is stable (Jacobian method) if *a*_*K*_ = *a*_2_ = *b*_2_ − *c* < 0, {*μK*_0_, *μK*_0_, 0, 0}, that is stable if *a*_*K*_ = *a*_1_ = *b*_1_ − *c* > 0 and $e<e_{c}=\frac{d+\lambda b_{1}}{\mu K_{0}}$ and $\left\{\mu K_{0},\mu K_{0}\frac{e_{c}}{e},\frac{\mu K_{0}\left(b_{1}+f\right)}{\left(b_{1}+d+f\right)}\left(1-\frac{e_{c}}{e}\right),\frac{\mu K_{0}d}{\left(b_{1}+d+f\right)}\left(1-\frac{e_{c}}{e}\right)\right\}$ that is stable if *a*_*K*_ = *a*_1_ > 0 and *e* > *e*_*c*_.

Thus, the stability analysis reveals that the EV infection develops as long as there is a positive growth rate and the infection rate is larger than a critical value, *e*_*c*_. This is the same condition we obtained in terms of the basic reproduction number using the next generation method. As expected the larger the the recovery rate, *d*, diminishes the possibility of an Ebola outbreak. Also, if bats are born infected, λ = 0, or the resources, *K*_0_, decrease it favors Ebola infection. As mentioned above, the case λ = 0 seems unplausible according to the demography of infected cases in bats and we assume hereafter that λ = 1, i.e. bats are EV born-free. As for the role played by the interplay between resources and bats, notice that 0 ≤ *μ* ≤ 1; if either bats do not condition the amount of available resources, *γ* = 0, or the resources are quickly replenished, *γ* ≪ *r*, then *μ* ≃ 1. Therefore, notably, the consumption of resources by the bats effectively buffers EV infection. Note also that, if either bats consume rapidly the resources, *γ* ≫ *r* or the replenishment rate vanishes, *r* = 0, then *μ* ≃ 0 and the bat population, as well as the resources become extinct.

### The environmental pressure modulates EV infection

**Fig 2:**
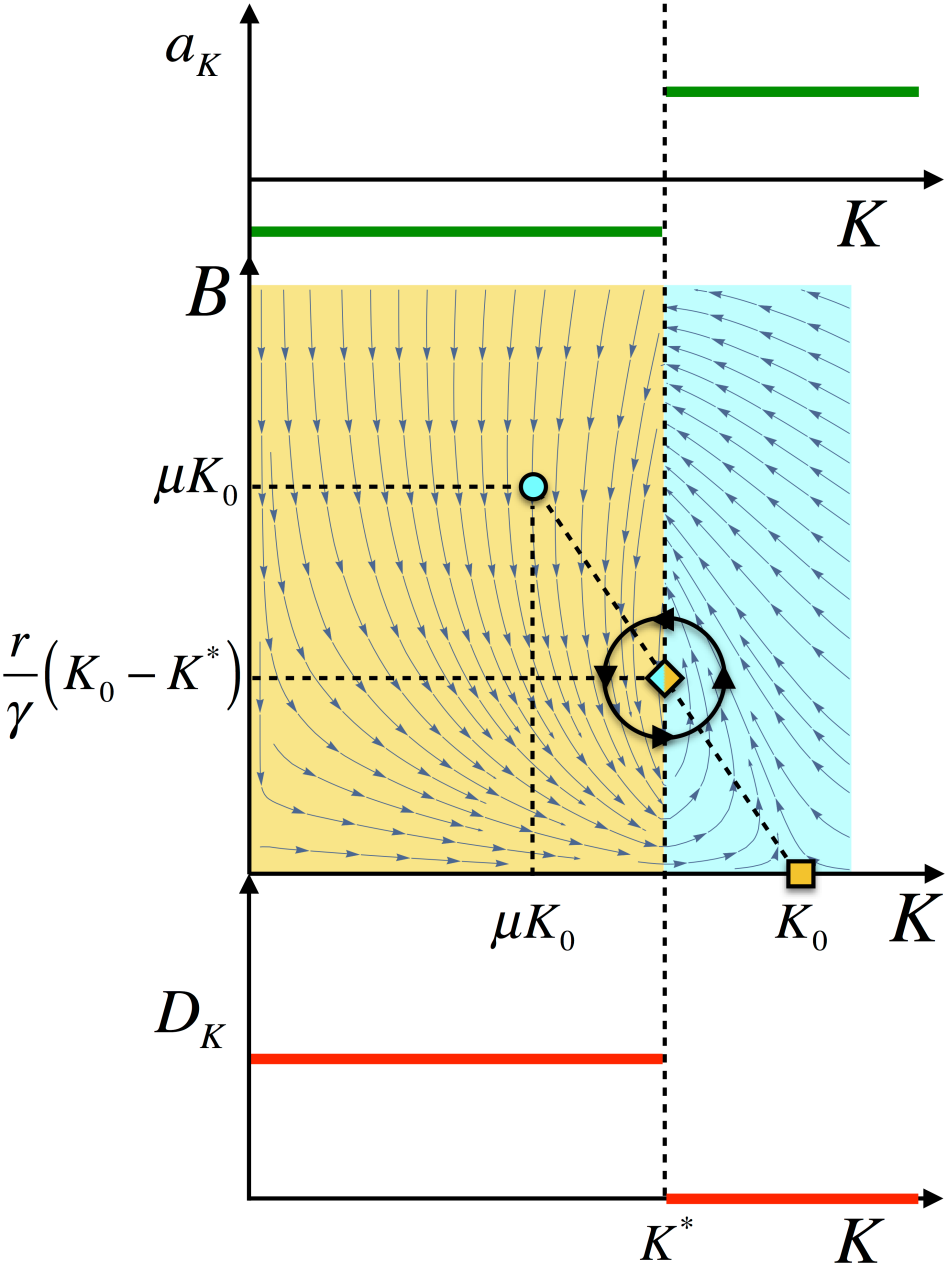
Functional relation of the growth rate, *a*_*K*_, (top), as a function of the resources, *K*. If the resources are below a critical value, K*, the growth rate is negative. If *K* > K* then *a*_*K*_ > 0. This leads to oscillations as shown in the *B* vs. *K* phase-space portrait (middle): in the blue region bats try to reach the blue attractor (circle) and in the yellow region the bats try to reach the yellow attractor (square). The chasing dynamics between these attractors with changing stability leads to an oscillatory behavior. Damped oscillations eventually relaxes to the induced attractor (diamond). We hypothesize that as response to the environmental pressure that conditions survival the bats migrate (bottom).

We explore the effects of the environmental pressure in the dynamics of the bats population assuming that if the available resources are below a certain value, K*, then the survival of the population is challenged. Thus, we assume that if *K* > K* then *b*_*K*_ = *b*_1_ > *c* (*a*_*K* > K*_ > 0) and if *K* < K* then *b*_*K*_ = *b*_2_ < *c* (*a*_*K* < K*_ < 0). According to the discussion above, in the former case the attractor corresponds to {*μK*_0_, *μK*_0_} while in the latter the attractor corresponds to {*K*_0_, 0}. Since the (stationary) amount of resources is bounded in the interval (*μK*_0_, *K*_0_), we assume that K* belongs to that interval. As shown in Fig 2, this mechanism compels bats and resources to chase attractors with an evanescent stability. This in turn induces an oscillatory behavior and a new stable attractor develops. We notice that if K* ∉ (*μK*_0_, *K*_0_) then the environmental pressure does not play any role since the induced attractor is unstable and either {*μK*_0_, *μK*_0_} or {*K*_0_, 0} are stable depending on the sign of the growth rate *a*_*K*_.

As for the location of the new attractor, we notice that $b_{K}=\lim_{n\to \infty }b_{1}+(b_{2}-b_{1})\frac{K^{{*}^{n}}}{K^{{*}^{n}}+K^{n}}$. By using this functional form of *b*_*K*_ instead of Heaviside step functions we avoid the difficulties derived by its non-continuous behavior. Hence, by solving Eqs (5) and (6) using this definition we found that,

$$\begin{array}{c} \left\{K^{st},B^{st}\right\}=\left\{K^{*}{\left(\frac{c-b_{2}}{b_{1}-c}\right)}^{\frac{1}{n}},\frac{\mu \left(K_{0}-K^{*}{\left(\frac{c-b_{2}}{b_{1}-c}\right)}^{\frac{1}{n}}\right)}{1-\mu }\right\} \end{array}$$ (11)

Thus, in the limit *n* → ∞, we obtain $\left\{K^{st},B^{st}\right\}=\left\{K^{*},\frac{\mu (K_{0}-K^{*})}{1-\mu }\right\}$. A stability analysis (Jacobian method) reveals that the chasing dynamics of the evanescent attractors is relaxational (the real part of the eigenvalues is negative). Consequently, the induced oscillations are not sustained but damped, i.e. a focus-like behavior, and the system eventually relaxes to this fixed point that is located in the pathway connecting the evanescent attractors. The same procedure can be implemented in the case of Eqs (1) and (4) and we obtain that EV-infected bats appear as long as $e>e_{c}^{*}=\frac{\left(1-\mu )(c+d\right)}{\mu (K_{0}-K^{*})}$. In that case the stationary attractor reads: $\left\{K^{st},B_{S}^{st},B_{I}^{st},B_{R}^{st}\right\}=\left\{K^{*},\frac{\mu (K_{0}-K^{*})}{1-\mu }\frac{e_{c}^{*}}{e},\frac{\mu (c+f)(K_{0}-K^{*})}{\left(1-\mu )(c+d+f\right)}(1-\frac{e_{c}^{*}}{e}),\frac{\mu d(K_{0}-K^{*})}{\left(1-\mu )(c+d+f\right)}(1-\frac{e_{c}^{*}}{e})\right\}$. Otherwise, if $e<e_{c}^{*}$, the stationary state is $\left\{K^{st},B_{S}^{st},B_{I}^{st},B_{R}^{st}\right\}=\left\{K^{*},\frac{\mu (K_{0}-K^{*})}{1-\mu },0,0\right\}$. In terms of population fractions the different states read: $f_{S}=\frac{e_{c}^{*}}{e}$, $f_{I}=\frac{1}{1+\in }(1-\frac{e_{c}^{*}}{e})$, and $f_{R}=\frac{\in }{1+\in }(1-\frac{e_{c}^{*}}{e})$ where $\in =\frac{d}{c+f}$. We can combine states *S* and *R* as healthy, *H*, such that: $f_{H}=\frac{e_{c}^{*}+\in e}{e(1+\in )}$.

**Fig 3:**
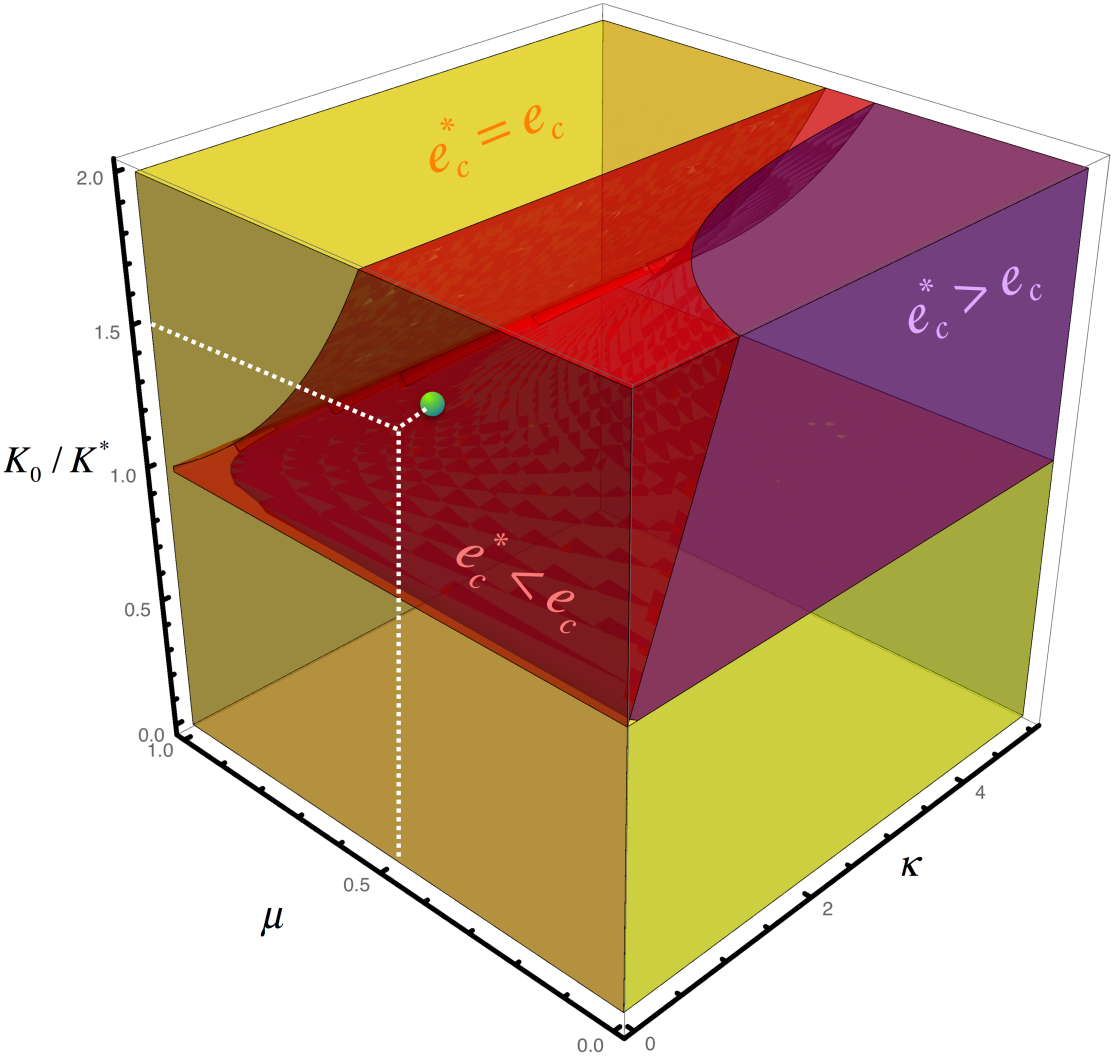
The combined effects of the environmental pressure and the feedback between the amount of resources and the population of bats on the modulation of the EV infection threshold can be summarized in a region plot as a function of the dimensionless parameters *κ*, *μ*, and *K*_0_/K*. In the red region the environmental pressure advances the infection, in the purple region the environmental pressure postpones the infection, finally, in the yellow region the infection threshold is unaffected. The green dot indicated the condition for our numerical simulations (see Table 1): *κ* = 17/28, *μ* = 1/2, and *K*_0_/K* = 3/2.

Interestingly, the results indicate that if K* ∈ (*μK*_0_, *K*_0_) then the environmental pressure can possibly either promote EV infection, if $e_{c}^{*}<e_{c}$, or hinder the EV infection, if $e_{c}^{*}>e_{c}$. The conditions $e_{c}^{*}\;≶\;e_{c}$ reduce to (1 + *κ*) / (1 + *μκ*) ≶ *K*_0_/K* where $\kappa =\frac{c+d}{a_{1}}$. Fig 3 shows, as a function of the dimensionless parameters *κ*, *μ*, and *K*_0_/K*, the regions for which the environmental pressure plays different roles.

We can further analyze the infection dynamics by calculating the basic reproduction number. In that case, the transmission and transition terms read,

V=c+d (12)

$$F=\frac{\mu \left(K_{0}-K^{*}\right)}{1-\mu }\left(e-\frac{\left|b_{1}+b_{2}-2c\right|}{2K^{*}}\right)$$ (13)

Hence,

$$\begin{array}{c} R_{0}=\frac{e}{e_{c}}-\frac{\left|b_{1}+b_{2}-2c\right|}{2K^{*}e_{c}} \end{array}$$ (14)

In our simulations, see Table 1, $R_{0}\approx 1.23$. As for the onset for infection, i.e. the condition $R_{0}>1$, it reduces to, *e* > *e*_*c*_ + |*b*_1_ + *b*_2_ − 2*c*|/(2K*). Thus the threshold for infection progagation, as estimated by the basic reproductive number, is more restrictive than the condition found using the stability analysis (see Discussion).

### Seasonality and climate variability: Sustained migration waves

**Fig 4:**
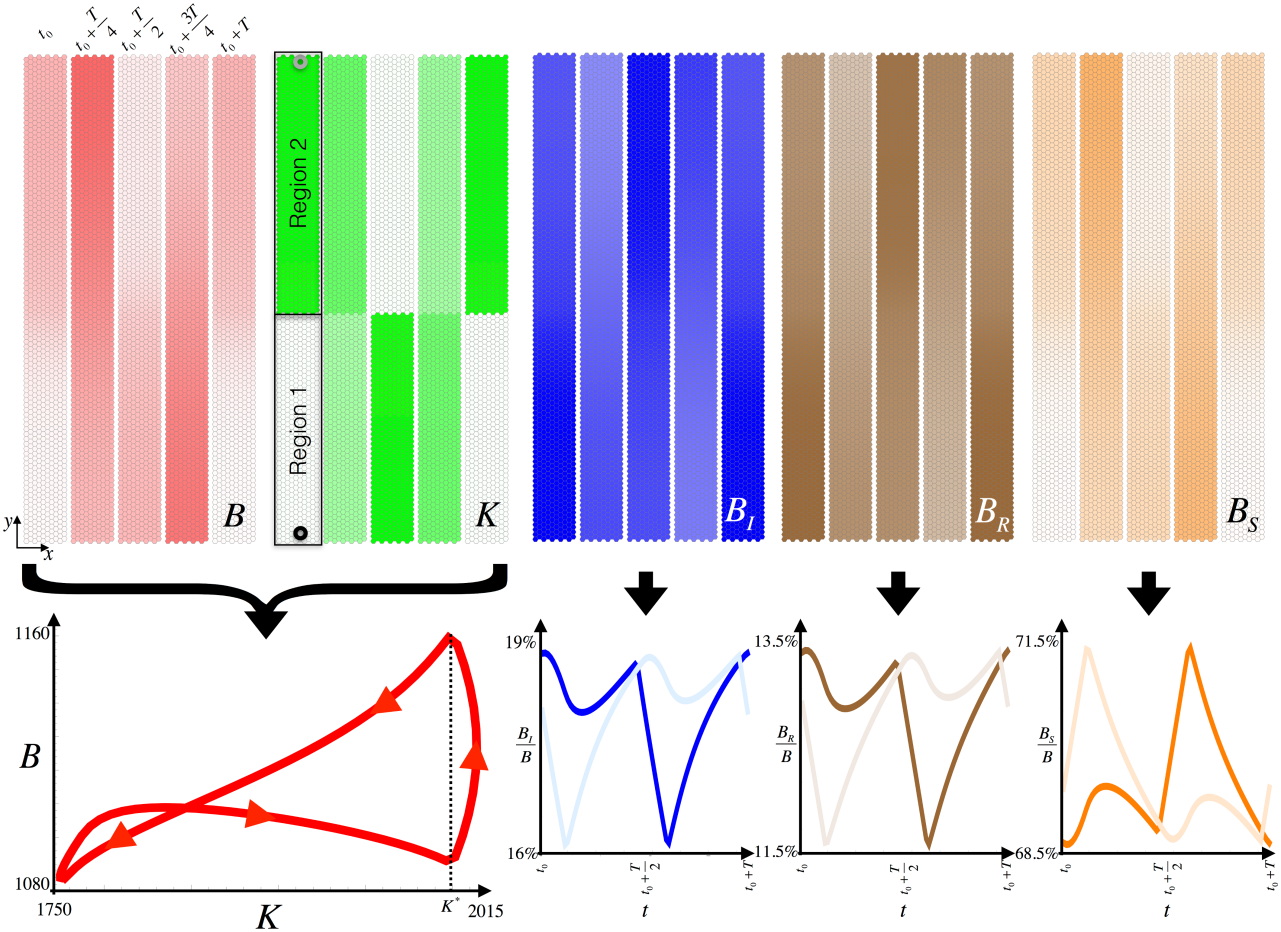
Form left to right, the top panels show the spatiotemporal oscillations of total bats (*B*), resources (*K*), infected bats (*B*_*I*_), recovered bats (*B*_*R*_), and susceptible bats (*B*_*S*_). The stripes of each top panel depict snapshots (density plots) of different time points during a period, *T*, of the stationary oscillations. In all cases the different time points as indicated in the first panel (*B*): *t*_0_, $t_{0}+\frac{T}{4}$, $t_{0}+\frac{T}{2}$, $t_{0}+\frac{3T}{4}$, and *t*_0_ + *T*. The second top panel (*K*) indicates the two spatial regions considered in our simulations (see Eq 15). Within each region a specific location is highlighted by a circle. Those indicate the locations for which the time series for the fractions $\frac{B_{I}}{B}$, $\frac{B_{R}}{B}$, and $\frac{B_{S}}{B}$ have been plotted: the dark (light) color time series correspond to the dark (light) circles in region 1 (2). The left bottom panel shows the stationary oscillations of the total bats during a period in the *B* vs. *K* phase space at any given location.

As for the spatiotemporal dynamics of our model, if the initial condition is space-dependent then mobility (diffusion) leads to transient migratory waves. Yet, if no further considerations are taken into account, every location reaches the same stationary state and mobility, eventually, does not play any role in the dynamics of infection. However, temperature, precipitation and other environmental indicators display periodicity and, in addition, environmental factors are also affected by the geographical location. As shown below these features, in combination with mobility terms, produced sustained migration waves. In our modeling approach we implement the seasonality and climate variability by considering the following, simple, spatiotemporal dependence of the bare carrying capacity, *K*_0_,

$$\begin{array}{c} K_{0}\left(r,t\right)=\left\langle K_{0}\right\rangle \left(1+\Gamma \text{cos}\left(\omega t+\Phi \left(r\right)\right)\right) \end{array}$$ (15)

where 〈*K*_0_〉 stands for an average carrying capacity, 0 < Γ < 1 the fractional variation that we assume constant, *ω* the seasonality temporal frequency, and Φ(**r**) is a phase that accounts for the spatial variability: different regions are subjected to the same periodic behavior of the resources but asynchronously. Here, we will consider the case of two well differentiated regions such that Φ(**r**) = *πδ*_**r**,**r′**_, where **r′** indicates all points that belong to one of the regions (see Fig 4).

Under these conditions, if *D*_*K*_ ≠ 0, traveling waves of migrating bats develop. As for *D*_*K*_, we hypothesize that bats migration must be coupled to environmental pressure: we argue that bats migrate if the lack of resources threatens their survival. In our model we implement this response to the environmental pressure by means of an non-homogeneous diffusion coefficient: *D*_*K*_ = *D* ≠ 0 if *K* < K* and *D*_*K*_ = 0 otherwise (see Fig 2).

Fig 4 shows the oscillatory behavior of bats population as a function of space and time and reveals their migratory dynamics chasing resources. With the parameters used in our simulations the percentage of EV infected bats oscillates between ∼16% and ∼19% depending on the season; the season when the amount of resources is minimal/maximal having the largest fraction of EV infected/healthy bats.

## Discussion

From the point of view of the required framework to study the outbreaks and spillovers associated to zoonotic diseases, one possible approach is to use models able to deal with the complex behavior arising from the interactions of individual units (human, animal,…). Moreover, in the particular case of Ebola, understanding the various levels of complexity of the problem ultimately requires anthropological and ecological considerations. Thus, the high mobility of humans in Africa through porous borders, cultural practices, socioeconomic conditions, or the high diversity of Ebola zoonotic carriers have been recognized as important elements [47]. Rule-Agent based models can provide such detailed level of description and establish predictive correlations among relevant factors. However, to identify fundamental mechanisms is difficult within such framework. Another option is to use a reductionist modeling approach, less precise from the viewpoint of the predictive character, but able to pinpoint precisely underlying mechanisms. Herein we have chosen this second alternative by using a compartmental model to address a specific question: how do the interplay between seasonality, climate variability, and environmental pressure drive the migration patterns of bats and the Ebola infection dynamics in their population?

Our model first assumes a bidirectional coupling between the available resources, effectively described by means of a carrying capacity variable, and different states of the bats population such that they reshape their respective equilibrium values. Environmental pressure is taken into account by hypothesizing a threshold below which the survival of the population is challenged. We have shown that environmental pressure induces new equilibrium states and an oscillatory behavior. Moreover, our results reveal that the environmental pressure modulates the population dynamics and, given an infection rate, can either promote or hinder the infection levels depending on the balance of three factors: the ratio between the rate of resources consumption by bats and the replenishing rate, the value of the carrying capacity threshold setting environmental pressure with respect to the “bare” resources, and a dimensionless parameter that accounts for the growth rate of the bat population and their ability to recover from infection. Finally, when seasonality and variability are considered as factors regulating the available resources and we assume conditional migration as a response mechanism to the population survival, we have shown that sustained waves of migratory bats develop. The migratory waves are correlated with the dynamics of the resources and our model suggests that infection is more probable when resources are low. The reason for this behavior is an increased competition for resources during those periods.

As a matter of discussion, here we have considered a minimal approach in terms of the possible states of infection: susceptible, infected and recovered. We have included a recovered state based on serological data of bats infected with Marburg filovirus [23]and physiological studies about Ebola infected bats [15]–[17]. We argue that if the death rate of bats is no changed because of the infection and adults are not the population sector with the largest number of infection cases then a recovery state can explain these observations. We acknowledge that this hypothesis could be eventually rebutted or that other serological states are possible (e.g. inmune, exposed). We notice that by presenting a more complex model with additional infection states we would rely on un-tested hypotheses and also on the estimation of additional parameters that are unknown at this point. Also, it is worth noting that some of the main conclusions of our study do not depend on this: the dynamics that set the basic interplay between the bat population and the environment, as given by Eqs (5) and (6), is independent of having a recovery state. In addition, the expressions that determine the concentration of different states or the phase diagram plot (Fig 3) are valid even in the case that the recovery rate is zero.

Our results also reveal an interesting effect in regards of the reproduction number due to the environmental pressure. In the absence of the environmental pressure, the condition found for the onset of infection is the same regardless whether it is obtained either using the Jacobian approach (stability analysis of the stationary infection-free solution) or through the next generation method (basic reproduction number). However, when the birth rate is a function of the available resources (environmental pressure) then the threshold obtained depends on the approach. This issue has been pointed out in other studies [41, 48]: while a linear stability analysis (Jacobian method) reveals the stability condition of the disease-free state it does not necessarily provides a value of $R_{0}$ that is biologically meaningful. Likewise, the threshold condition found using the basic reproduction number does not necessarily imply an asymptotic stability condition of the disease-free state.

A number of studies have shown that in the context of population dynamics and infectious diseases the diffusion may play a critical role and leads to the emergence of patterns [49–51]. The underlying mechanism is based on the Turing instability that relies on the existence of distinct diffusion scales for different species [52]. In the case of the Ebola infection in bats, data does not suggest that their physiology is altered due to the infection and, consequently, we have not consider different migratory capabilities depending on their infection status. As a result, the emergence of spatial patterns of infection can be discarded in our model and diffusion plays merely an homogenizing role that spreads EV infection when the environmental pressure forces migration. However, we notice that possible extensions of our model might consider a multiple species approach given that the zoonotic niche of Ebola is larger than originally expected [12]. In that case, the migratory capabilities would be certainly different depending on the host and this would open the possibility of finding a pattern emergent behavior.

Our study can be relevant to predict hot spots of Ebola outbreaks in space and time and may also suggest conservation policies to mitigate the risk of spillovers. This depends, ultimately, on the possibility of calibrating our model with the right, realistic, parameters and points out the importance of quantitative ecology and climatology approaches. As for possible extensions of this study, our results suggest the importance that the role played by stochasticity in climate and seasonality since large fluctuation events, while having low probability, may lead to a significant increase in the infected population of bats and therefore of an Ebola outbreak. Work to explore these factor is in progress.
